# The Enterococcus Cassette Chromosome, a Genomic Variation Enabler in Enterococci

**DOI:** 10.1128/mSphere.00402-18

**Published:** 2018-11-07

**Authors:** A. Sivertsen, J. Janice, T. Pedersen, T. M. Wagner, J. Hegstad, K. Hegstad

**Affiliations:** a Research Group for Host-Microbe Interactions, Department of Medical Biology, Faculty of Health Sciences, UiT-The Arctic University of Norway, Tromsø, Norway; b Norwegian National Advisory Unit on Detection of Antimicrobial Resistance, Department of Microbiology and Infection Control, University Hospital of North Norway, Tromsø, Norway; c Department of Microbiology and Infection Control, University Hospital of North Norway, Tromsø, Norway; University of Iowa

**Keywords:** *Enterococcus faecium*, enterococci, mobile genetic element, serine recombinase, *ccrAB_Ent_*, SCC*mec*

## Abstract

Enterococcus faecium has a highly variable genome prone to recombination and horizontal gene transfer. Here, we have identified a novel genetic island with an insertion locus and mobilization genes similar to those of staphylococcus cassette chromosome elements SCC*mec*. This novel element termed the enterococcus cassette chromosome (ECC) element was located in the 3′ region of *rlmH* and encoded large serine recombinases *ccrAB* similar to SCC*mec*. Horizontal transfer of an ECC element termed ECC::*cat* containing a knock-in *cat* chloramphenicol resistance determinant occurred in the presence of a conjugative *rep*_pLG1_ plasmid. We determined the ECC::*cat* insertion site in the 3′ region of *rlmH* in the E. faecium recipient by long-read sequencing. ECC::*cat* also mobilized by homologous recombination through sequence identity between flanking insertion sequence (IS) elements in ECC::*cat* and the conjugative plasmid. The *ccrAB_Ent_* genes were found in 69 of 516 E. faecium genomes in GenBank. Full-length ECC elements were retrieved from 32 of these genomes. ECCs were flanked by *attR* and *attL* sites of approximately 50 bp. The *attECC* sequences were found by PCR and sequencing of circularized ECCs in three strains. The genes in ECCs contained an amalgam of common and rare E. faecium genes. Taken together, our data imply that ECC elements act as hot spots for genetic exchange and contribute to the large variation of accessory genes found in E. faecium.

**IMPORTANCE** Enterococcus faecium is a bacterium found in a great variety of environments, ranging from the clinic as a nosocomial pathogen to natural habitats such as mammalian intestines, water, and soil. They are known to exchange genetic material through horizontal gene transfer and recombination, leading to great variability of accessory genes and aiding environmental adaptation. Identifying mobile genetic elements causing sequence variation is important to understand how genetic content variation occurs. Here, a novel genetic island, the enterococcus cassette chromosome, is shown to contain a wealth of genes, which may aid E. faecium in adapting to new environments. The transmission mechanism involves the only two conserved genes within ECC, *ccrAB*_Ent_, large serine recombinases that insert ECC into the host genome similarly to SCC elements found in staphylococci.

## Abstract (precis)

Enterococcus faecium is a bacterium found in a great variety of environments, ranging from the clinic as a nosocomial pathogen to natural habitats such as mammalian intestines, water, and soil. They are known to exchange genetic material through horizontal gene transfer and recombination, leading to great variability of accessory genes and aiding environmental adaptation. Identifying mobile genetic elements causing sequence variation is important to understand how genetic content variation occurs. Here, a novel genetic island, the enterococcus cassette chromosome, is shown to contain a wealth of genes, which may aid E. faecium in adapting to new environments. The transmission mechanism involves the only two conserved genes within ECC, *ccrAB*_Ent_, large serine recombinases that insert ECC into the host genome similarly to SCC elements found in staphylococci.

## INTRODUCTION

Enterococci are a public health concern as a common cause of hospital-associated infections and a burden to patient morbidity and mortality. They have acquired antimicrobial resistance mechanisms toward many currently available antibiotics through horizontal gene transfer (HGT) of mobile genetic elements (MGEs) (1–4). They are also able to survive a broad range of environments and environmental stressors to which other bacteria succumb (5, 6). Enterococci contain a broad diversity of large integrative conjugative elements (ICEs) and nonconjugative genomic islands (GIs) believed to contribute to their genomic diversity (7).

The staphylococcus cassette chromosome element SCC*mec* is a GI in Staphylococcus aureus harboring the *mecA* gene providing resistance toward beta-lactams (8). Movement of SCC*mec* occurs by the serine recombinases CcrA and CcrB that recognize a specific attachment site (*attB*) in the 3′ region of *rlmH*, a conserved tRNA methyltransferase gene in S. aureus (9, 10), and a corresponding attachment site (*attSCC*) on the circularized SCC*mec* intermediate. CcrAB use these sites to integrate SCC*mec*, after which *attL* (5′) and *attR* (3′) sites are generated as excision sites on either end of the element (9, 11, 12). SCC*mec* elements show a large degree of diversity in the gene content in both S. aureus (13) and in other species within the *Staphylococcus* genus (14–16). HGT of SCC*mec* between staphylococci has been observed during antimicrobial therapy (17), in the lab using bacteriophages as transfer vehicles (18, 19), and by conjugation after SCC*mec* integration by homologous recombination of IS elements into a staphylococcal conjugative plasmid *in vitro* (20).

Orthologues to the S. aureus *ccrAB* genes have been found by screening a collection of several species of the *Enterococcus* genus (21). These *ccrAB_Ent_* genes were expressed as a bicistronic mRNA (21) in reference strain Enterococcus faecium DO (22).

Here, we demonstrate the mobility of the novel genetic island enterococcus cassette chromosome (ECC) in E. faecium. ECC shares insertion site and movement by large serine recombinases with SCC*mec*. ECCs are present in 9% of available E. faecium genomes in the NCBI database, and their gene content is highly variable. We postulate that ECCs act as gene traffickers between the enterococcal chromosomes.

## RESULTS AND DISCUSSION

### ECC::*cat* was successfully transferred between strains by the help of a conjugative *rep*_pLG1_ megaplasmid.

UWECC::*cat* is a clinical plasmid-cured E. faecium strain with a knocked-in selectable marker, the chloramphenicol resistance-encoding gene *cat* immediately downstream of *ccrAB_Ent_*. Filter mating experiments using strain UWECC::*cat* without a helper plasmid failed to produce transconjugants with ECC::*cat* within the detection limits (10^−10^ to 10^−9^ transconjugants/donor cell). To mobilize ECC::*cat* into recipient strain BM4105-RF, a conjugation apparatus was provided by filter mating a 298-kb *rep*_pLG1_ megaplasmid into UWECC::*cat* via BM4105-RF from clinical isolate K60-19 (Table 1; see also Fig. S1 in the supplemental material) (23). The *rep*_pLG1_ megaplasmid contains a type IV secretion system (T4SS), an *aac*(*6’*)*Ie-aph*(*2”*)*Ia* gentamicin resistance selection determinant, and belongs to the RepA_N family which previously has been shown to mobilize large chromosomal stretches of DNA in E. faecium (24, 25). Transconjugants occurred at frequencies of 3 × 10^−7^ per donor in UWECC::*cat rep*_pLG1_ × BM4105-RF filter mating experiments.

**TABLE 1:** Bacterial experimental strains and plasmids

Species and strain	Plasmid	Relevant resistance characteristic(s) [gene(s)]^*a*^	Relevant description	Type of sequence data	Reference	GenBank accession no.
E. faecium strains						
UW1551			ECC-containing clinical isolate		52	
UWΔp			UW1551 cured of most plasmids		This study	
UWECC::*cat*		Chl^r^ [*cat*]	Plasmid-cured UW1551 with *cat* resistance marker inserted in ORF1 next to *ccrAB_Ent_*		This study	
K60-19	*rep*_pLG1_	Gen^r^ [*aac*(*6*′)*Ie-aph*(*2''*)*Ia*]	Clinical *rep*_pLG1_ plasmid donor with many other plasmids		23	
BM4105-RF *rep*_pLG1_	*rep*_pLG1_	Rif^r^, Fus^r^, Gen^r^ [*aac*(*6*′)*Ie-aph*(*2''*)*Ia*]	*rep*_pLG1_ plasmid donor containing only this plasmid		This study	
UWECC::*cat rep*_pLG1_	*rep*_pLG1_	Chl^r^ [*cat*], Gen^r^ [*aac*(*6*′)*Ie-aph*(*2''*)*Ia*]	Donor UWECC::*cat* with *rep*_pLG1_	PacBio	This study	NMZL01000001.1, NMZL01000002.1, NMZL01000003.1
BM4105-RF		Rif^r^, Fus^r^	Recipient	Nanopore and Illumina combined	54	CP030110.1
BMECC::*cat*	*rep*_pLG1_	Chl^r^ [*cat*], Rif^r^, Fus^r^, Gen^r^ [*aac*(*6*′)*Ie*-*aph*(*2''*)*Ia*]	Transconjugant containing ECC::*cat* on BM4105-RF chromosome	PacBio	This study	NMZK01000001.1, NMZK01000002.1
BM pECC::*cat*	*rep*_pLG1_ECC::*cat*	Chl^r^ [*cat*), Rif^r^, Fus^r^, Gen^r^ [*aac*(*6*′)*Ie*-*aph*(*2''*)*Ia*]	Transconjugant containing ECC::*cat* on plasmid	PacBio	This study	NMZJ01000001.1, NMZJ01000002.1

E. coli strains						
pTEX5500ts		Chl^r^ [*cat*], Gen^r^ [*aph*(*2''*)-*Id*]	Shuttle plasmid, temperature sensitive in Gram-positive host		53	
pORF1a		Chl^r^ [*cat*], Gen^r^ [*aph*(*2''*)-*Id*]	pTEX5500ts with cloned ORF1 fragment upstream of the *cat* gene		This study	
pORF1b		Chl^r^ [*cat*], Gen^r^ [*aph*(*2''*)-*Id*]	pTEX5500ts with cloned ORF1 fragments flanking the *cat* gene		This study	

a The r superscript indicates resistance. Drugs are abbreviated as follows: Chl, chloramphenicol; Gen, gentamicin; Rif, rifampin; Fus, fusidic acid.

10.1128/mSphere.00402-18.4 FIG S1 Filter mating scheme showing the transfer of mobile genetic elements from donors to recipients. A *rep*_pLG1_ plasmid (gray ring) was transferred from clinical isolate K60-19 (green) into BM4105-RF (orange, top). This plasmid was then transferred into UWECC::*cat* (blue) and back again into BM4105-RF with the hitchhiking ECC::*cat* (black curled line). BM4105-RF transconjugants (orange, bottom) containing ECC::*cat* in the chromosome (right) or on the plasmid with *rep*_pLG1_ (left). Download FIG S1, PDF file, 0.7 MB. Copyright © 2018 Sivertsen et al. 2018 Sivertsen et al. This content is distributed under the terms of the Creative Commons Attribution 4.0 International license.

We also obtained horizontal transfer of ECC::*cat* by the aid of five other *rep*_pLG1_ megaplasmids from clinical E. faecium strains (results not shown), confirming that they are vehicles for mobilization of genetic elements in E. faecium.

### ECC::*cat* was inserted into the recipient chromosome in an SCC*mec*-like fashion.

**FIG 1:**
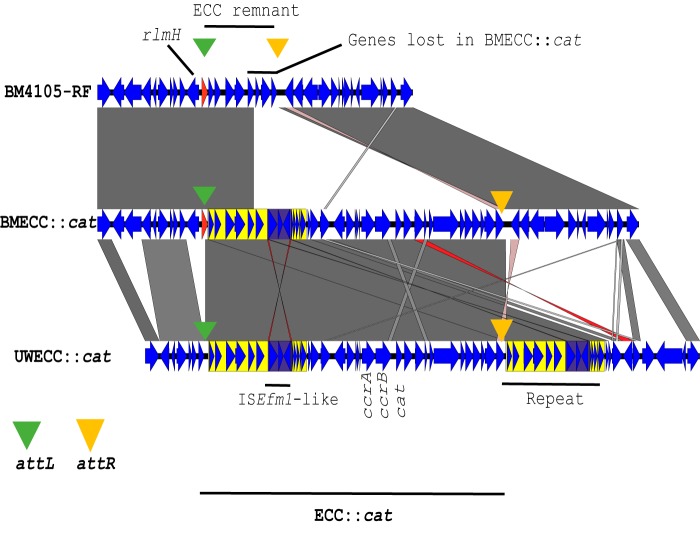
Pairwise alignment showing the genetic organization of chromosomal integration of ECC::*cat*. The insertion region in recipient BM4105-RF (top) is aligned with its transconjugant BMECC::*cat* (middle) after horizontal transfer from UWECC::*cat* (bottom). The *ccrAB_Ent_* genes and *cat* knock-in location are highlighted in UWECC::*cat*. Green and orange triangles show the locations of *attL* and *attR* sites, respectively. The *rlmH* gene is drawn in red. Ten-kilobase direct repeats in UWECC::*cat* are highlighted in yellow, and the IS*Efm1* element is highlighted in purple.

E. faecium UWECC::*cat*, recipient BM4105-RF, and two transconjugants were long read sequenced to resolve genomic structures and identify insertion sites of ECC::*cat*. A 32-kb ECC element was inserted chromosomally downstream of *rlmH* in the transconjugants BMECC::*cat*, flanked by direct repeat regions representing *att* sites (Fig. 1 and Table S1). BM4105-RF contains an ECC remnant, as an *attL* site is contained within *rlmH* and an *attR* site could be identified downstream. Three genes near the *attR* site had been lost in the transconjugant compared to recipient BM4105-RF (Fig. 1). The likely explanation for the organization of the ECC::*cat* chromosomal region in transconjugant BMECC::*cat* is excision and loss of the recipient’s ECC remnant and subsequent replacement with ECC::*cat*.

10.1128/mSphere.00402-18.1 TABLE S1 Metadata of strains and ECCs in E. faecium genomes. Download Table S1, XLSX file, 0.02 MB. Copyright © 2018 Sivertsen et al. 2018 Sivertsen et al. This content is distributed under the terms of the Creative Commons Attribution 4.0 International license.

**FIG 2:**
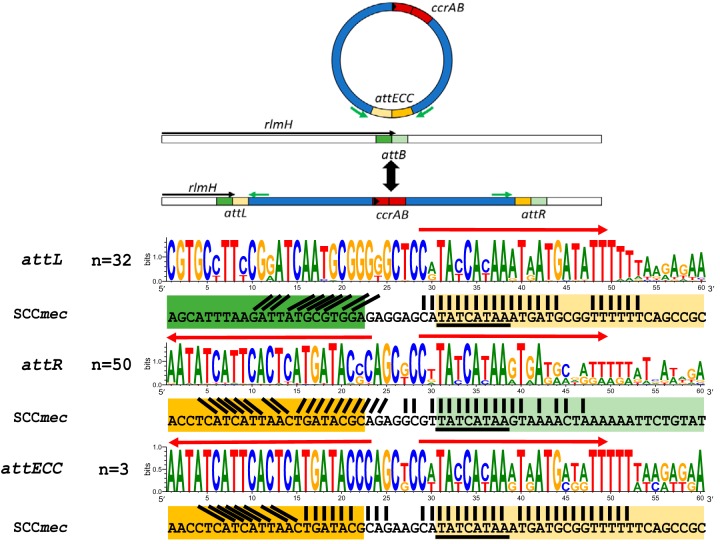
ECC movement and MEME motifs of *att* site sequences in ECC elements. (A) Schematic view of the circular intermediate of ECC and ECC integrated into the chromosome. The colors of *att* site halves in the figure and between MEME motifs and SCC*mec* sequences are identical. The locations of circularization PCR primers are shown with green arrows. (B) MEME motifs of enterococcal putative ECC *attL* and *attR* sites (colored letters) and *att* sites from S. aureus N315 (black letters), with central *ccrAB* recognition motifs underlined. Imperfect inverted repeats in *att* sites are shown by red arrows. The numbers of sequences used to create the MEME motifs are shown on the left of the sequences.

The identified direct repeats of approximately 50 nucleotides flanking ECC::*cat* showed similarity to direct repeats found in SCC*mec*-containing S. aureus N315 (Fig. 2), and thus represent *att* sites compatible with *ccrAB_Ent_*-mediated specific excision and insertion. The repeats contained a conserved central motif (5′-TATCATAA-3′) identical to SCC*mec att* sites.

### Excision of ECC.

ECC is expected to circularize during excision, as is observed in SCC*mec*. Circularization PCRs of ECC::*cat* and ECC elements from E. faecium DO and K59-68 (Fig. 2, primers in green arrows) were Sanger sequenced, showing circularization of ECC in these strains (*attECC* sequences in Table S1). The consensus sequences in Fig. 2 show how the *attECC* and *attR* sites contain inverted repeats (red arrows), creating a dyad symmetry characteristic of serine recombinase *att* sites (26).

### ECC elements in enterococcal genomes.

In order to evaluate the presence of ECCs in enterococci, 1,478 enterococcal genomes downloaded from NCBI, including three PacBio-sequenced strains in our own collection (UWECC::*cat*, K59-68, and 9-F-6) were analyzed by BLASTn searches for *ccrAB_Ent_*. The *ccrAB_Ent_* genes spread sporadically throughout the *Enterococcus* genus, as BLAST hits were found in E. faecium, E. faecalis, E. durans, E. hirae, and E. mundtii in addition to five enterococci without species designations (Table 2).

**TABLE 2:** Number of enterococcal genomes analyzed and positive for *ccrAB_Ent_*

Species	No. of genomes analyzed	No. of genomes positive for *ccrAB_*Ent*_*
E. faecium	516	69
E. faecalis	677	4
E. durans	10	4
E. hirae	34	8
E. mundtii	20	1
*Enterococcus* sp.	221	5

Total	1,478	91

**FIG 3:**
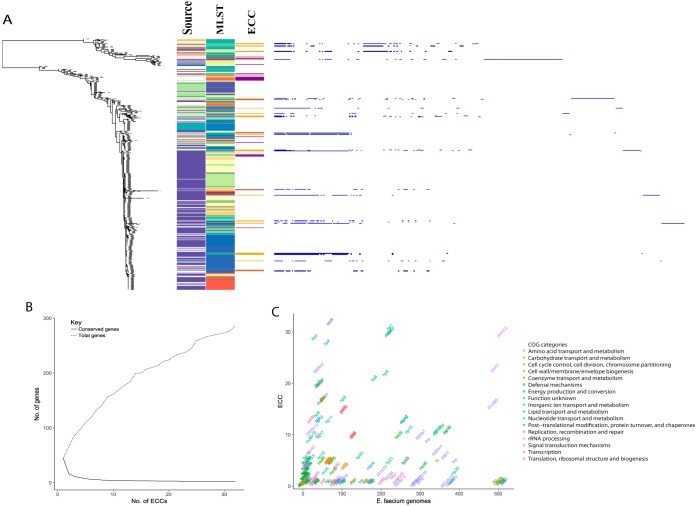
Presence of ECC in enterococcal genomes, pan-genome analyses of genes present in ECC. (A) Phandango-generated overview of WGS tree of 516 E. faecium genomes created by parsnp, with annotated MLST profiles as shown by colors. The presence of ECC elements (yellow for full elements and purple for *ccrAB_Ent_*-positive, fragmented assembly) and source of isolation (violet for human, turquoise for lab strain, green for animal, yellow for environment, orange for food) are shown by different colors. To the right, pan-genome plot in blue showing genes in ECCs, sorted vertically by the position of ECC-positive strain in phylogeny and horizontally by gene prevalence with the most abundant genes to the left. (B) Graph showing accumulating number of accessory genes and conserved genes in ECCs. (C) Scatter plot of genes annotated by eggNOG, plotted in coordinates corresponding to occurrences of gene in ECCs (*y* axis) and E. faecium genomes (*x* axis), with colors corresponding to the assigned cluster of orthologous group (COG).

We decided to analyze elements in E. faecium, as they contained the most *ccrAB_Ent_*-positive strains (Fig. 3A, Table 2, and Table S1). It was of interest to see whether ECC was enriched in specific lineages or environments. As determined by the E. faecium whole-genome sequence (WGS) phylogeny and metadata (shown for complete ECCs in Fig. S2 and Table S1), the *ccrAB_Ent_*-containing isolates are found in both commensal and nosocomial lineages and originate from both clinical, farm animal, and commensal sampling sites without apparent preference.

10.1128/mSphere.00402-18.5 FIG S2 Gene synteny and metadata among ECC elements in E. faecium and one *E. durans* sorted according to *ccrAB_*Ent*_* phylogeny. To the left is a RAxML maximum likelihood tree of the *ccrAB*_Ent_ genes aligned with Mafft, with 100 bootstraps. Isolates are colored by species or clade as follows: *E. durans* (black), E. faecium commensal clade (blue), and E. faecium clinical/farm animal clade (red). In the middle, metadata are shown as apparent from the Patric database (white indicates no available information). To the right is a Mauve alignment of ECC elements with conserved regions marked with individual local collinear block colors. Download FIG S2, PDF file, 2.3 MB. Copyright © 2018 Sivertsen et al. 2018 Sivertsen et al. This content is distributed under the terms of the Creative Commons Attribution 4.0 International license.

Complete ECC elements were identified by two criteria: *ccrAB_Ent_* located downstream of *rlmH* and the presence of identifiable *attL* and *attR* sites. The *ccrAB_Ent_* genes were often located on small contigs and/or near contig ends in many short-read-based assemblies thus impairing analysis of the up- and downstream regions. Thirty-two complete or scaffolded ECC elements with *ccrAB_Ent_* and both *attL* and *attR* could be identified in E. faecium.

### ECC putative *attL* and *attR* sites are conserved 50-bp sequences.

To evaluate *att* site conservation among ECC elements, we searched for *att* sites in ECC-containing strains with BLASTn-short (Table S1) and concatenated all identified *att* site regions into MEME motifs (Fig. 2B). The putative *attL* and *attR* sites consist of 50-bp direct repeats, containing inverted repeats in *attR* and in *attECC* after ECC excision/circularization (Fig. 2B, red arrows). The *att* sites from S. aureus strain N315 (12) were included for comparison. A conserved (5′-TATCATAA-3′) motif in SCC*mec* is conserved in ECC *att* sites and is also partly present on the complementary strand (5′-ATGATA-3′) within the inverted repeat in *attR* and *attECC* (Fig. 2). According to Wang et al. (12), the only essential nucleotide capable of completely abrogating SCC*mec* CcrAB function if substituted is the C surrounded by the TAT/ATA palindrome (5′-TATCATAA-3′). This nucleotide was conserved in all ECC *attL* and *attR* sequences.

To investigate the number of *att* sites present in each genome, *att* sites were queries in BLASTn-short analyses. This consistently resulted in less than five hits per genome and *att* sites most often located near *ccrAB_Ent_* in circularized genomes. Multiple *attR* sites could be found in 16 of 32 ECCs (Table S1), as has also been observed in S. aureus strains containing complex SCC elements, see Wang et al. (12) and references therein. One isolate (GCA_000321805/EnGen0001) had ECC on two contigs, of which one spanned both *att* sites. However, tandem ECCs with multiple *ccrAB_Ent_* genes were not observed directly.

### ECCs are highly variable in gene content.

After identifying 32 ECC elements in enterococci, the basal features of size and content were analyzed. The sizes of the ECCs varied from 21 kb to 78 kb (Table S1), with an average of 42 kb. There were on average 42 ORFs in each ECC, and the largest contained 92 ORFs.

A Roary pan-genome analysis was done to evaluate the gene content of ECCs and identified 283 gene clusters. Most genes were present in one ECC or in a few ECCs (Fig. 3A and Table S2). This is also reflected in the core/pan-genome plot (Fig. 3B), which shows a limited number of shared genes (*ccrA*, *ccrB*, and insertion gene *rlmH*).

10.1128/mSphere.00402-18.2 TABLE S2 Genes found as part of ECCs in E. faecium. Download Table S2, XLSX file, 0.1 MB. Copyright © 2018 Sivertsen et al. 2018 Sivertsen et al. This content is distributed under the terms of the Creative Commons Attribution 4.0 International license.

We hypothesized that the most abundant genes in ECCs were specific to this element and would not be present in strains without ECC. A BLAST database of representative genes clustered in ECC as determined by Roary was created and used as the basis to search for ECC genes among the 516 E. faecium genomes investigated. Interestingly, many of the ECC genes are common in E. faecium genomes, but not necessarily as part of ECCs (Fig. 3C).

The ECC insertion locus *rlmH* resides in >99% of the enterococcal strains and therefore could serve as an entry point for ECCs in most enterococci. The analyses showed two alleles of this gene with less than 75% DNA identity. Both *rlmH1* and *rlmH2* contained the *attL* site. The locations of the 283 ECC genes within circularized genomes (*n* = 26) were plotted to investigate the locations of these genes in E. faecium genomes relative to *rlmH*. These genes were found located throughout the whole genome in strains both with and without ECC elements (Fig. S3). This finding either supports that gene synteny conservation in E. faecium is limited or that ECCs may acquire gene cargo with limited conservation with regard to the position in the chromosome. Often, ECC-associated genes are enriched in the vicinity of *rlmH*, possibly representing ECC remnants or showing that ECCs are prone to engulf neighboring DNA. IS elements and transposases are found in abundance within ECCs and are likely carriers of genetic cargo entering ECCs by composite transposition or by representing homologous recombination sites between IS elements in ECCs and other genomic regions.

10.1128/mSphere.00402-18.6 FIG S3 Locations of ECC genes in E. faecium genomes. All genes found in ECCs except genes annotated to encode transposon units (indicated with gray letters in Data Set S2) were plotted according to genomic position in closed E. faecium genomes found in the NCBI databases. Genes clustered around positions 250000 to 300000, as indicated by the blue lines. Download FIG S3, PDF file, 0.3 MB. Copyright © 2018 Sivertsen et al. 2018 Sivertsen et al. This content is distributed under the terms of the Creative Commons Attribution 4.0 International license.

### ECC gene content may vary by ecological background.

The gene synteny of the 32 ECCs was assessed via a Mauve alignment. Fifty-four percent of the ECC genes were unique to only one ECC (Fig. 3C and Table S2) and tended to be connected within particular local colinear blocks (LCBs) (Fig. S2), thus representing independent genetic acquisitions. Strain habitat and phylogenetic proximity influence LCB content, as there is more variability between phylogenetic clades than within the clades, and ECCs in isolates from similar origins share more LCBs (Fig. S2). These observations indicate that ECC elements have a role in enterococci similar to that of SCC*mec* in staphylococci where the surrounding regions have been described as sequence variation “hot spots” (13, 27).

### Notable functions of genes enriched in ECC elements.

Mir-Sanchis et al. (28) characterized conserved hypothetical genes in SCC*mec*, containing domains with unknown functions DUF 927, DUF 950, DUF 960, and DUF 1643. Among these, DUF 960 (*n* = 30) and DUF 927 (*n* = 20) were found in ECCs (Table S2) including ECC::*cat*, which supports the idea that these ORFs may encode central unknown functions in both SCC*mec* and ECC. DUF 927 is predicted to encode a helicase, which implies autonomous replication of SCC*mec* in its circular state.

Genes associated with carbohydrate transport and metabolism were enriched among ECC genes and largely consisted of phosphotransferase systems (PTS) (Table S2). PTS genes associated with increased virulence such as *ptsD* encoding the PTS IID subunit which has been implicated in improved intestinal colonization during antimicrobial treatment (29) or the *bepA* gene encoding PTS permease implicated in endocarditis and biofilm formation (30) were not found.

Of interest, many ECC elements contained defense system-related genes (Table S2). Mostly, they were identified as *hsdR*, *hsdS*, and *hsdM* genes, which when all are present, encode a functional EcoKI type I restriction/modification (R/M) system. EcoKI has been observed in staphylococcal SCC elements and is thought to contribute to SCC persistence in its host (16, 31). Another R/M system (SfaNI) has previously been associated with *ccrAB_Ent_* in the single *ccrAB_Ent_*-positive E. faecalis strain (32), which further suggests that R/M systems are associated with cassette chromosome elements. Incomplete type I R/M systems (*n* = 13) occur more often than complete ones (*n* = 8) in ECC elements, which is surprising given that orphan methylases from type I R/M systems are rarely found (33) and should be inactive without *hsdS* (34).

One ECC harbors the tetracycline resistance gene *tetM* (Table S2) as part of a Tn*916*-like ICE. The reason why we do not see more of this resistance gene in other ECC elements may be that *tetM* is already present on a Tn*916*-like element which successfully transfers itself and inserts into random genomic sites with little sequence homology (35).

### Alternative mobilization by integration of ECC into the conjugative plasmid.

Species in which HGT frequently occur demonstrate “Russian doll”-like dissemination patterns of MGEs, permitting multiple pathways of movement within the cell as well as by HGT (36). Horizontal movement of large segments of chromosomal DNA has previously been shown in enterococci through conjugative plasmid cointegration of chromosomal DNA and subsequent integration into the recipient chromosome by recombination along homologous regions (24, 37).

**FIG 4:**
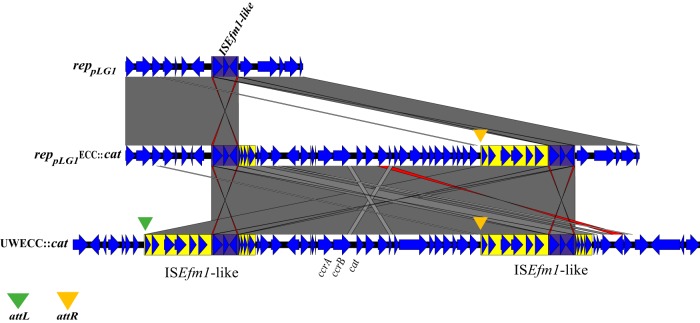
Pairwise alignment showing genetic organization of alternative *rep*_pLG1_ megaplasmid integration of ECC::*cat*. The *rep*_pLG1_ plasmid insertion region (top) is aligned with transconjugant BMpECC::*cat* plasmid (middle) after horizontal transfer from UWECC::*cat* (bottom). The IS*Efm1* element likely causing integration of UWECC::*cat* into the *rep*_pLG1_ megaplasmid is highlighted in purple. The *ccrAB_Ent_* genes and *cat* knock-in location are highlighted in UWECC::*cat*. Green and orange triangles show the locations of *attL* and *attR* sites, respectively.

Strain UWECC::*cat* contained a novel IS*982*-family IS*Efm1*-like element (purple in Fig. 4) within two 10-kb repeats (yellow) up- and downstream of the central region containing *ccrAB_Ent_*. This IS*Efm1*-like element has a size of 2427 bp. As seen in Fig. 4, the region transferred from UWECC::*cat* into the plasmid was bounded exactly by the two IS*Efm1*-like copies. Likely, plasmid integration was enabled by homologous recombination between IS*Efm1*-like elements. This hybrid plasmid was then transferred from strain UWECC::*cat* to BM4105-RF, resulting in BMpECC::*cat*, which contains an altered ECC::*cat* lacking *attL*.

In one closed chromosome (6E6/GCA_001518735), *ccrAB_Ent_* was present but was not located downstream of *rlmH*. Two *attR* sites were found downstream of the *ccrAB_Ent_* genes, but no *attL* site was found upstream. There are several IS elements up- and downstream of this *ccrAB_Ent_*, which could have contributed to alternative mobility.

The ECC of strain 9-F-6 harbors parts of Tn*6085* (38), a Tn*916*-like ICE, which may allow cotransfer of ECC with Tn*916*. Cotransfer of GIs by Tn*916* has previously been seen for the small GI MTnSag1 in Streptococcus agalactiae (39).

Mobilization of GIs by plasmids and ICEs has previously been shown (40, 41) and is dependent on compatibility between the hitchhiking GI and the conjugative element. Mobilizable GIs either encode a relaxase that is compatible with a type 4 coupling protein (T4CP) of a T4SS expressed by another conjugative MGE, or the T4SS may have a relaxase recognizing an *oriT* within the mobilizable GI to enable hitchhiking. Some relaxases show a less strict requirement for the base sequences within *oriT* sequences and can initiate transfer from a variety of sites (42, 43). The most likely ECC transfer mechanism is recognition of an *oriT* within the circular ECC by the *rep*_pLG1_ replication and conjugation apparatus.

Alternatively, ECC::*cat* encodes its own relaxase able to interact with the T4CP of the *rep*_pLG1_ T4SS apparatus. A gene determinant thought to engage in rolling-circle replication (*rep*) was detected in six of 32 investigated ECC elements, including isolate DO and isolate UWECC::*cat* used in the mobilization experiments. This is the same putative replication gene others have associated with *ccrAB_Ent_* (21, 28).

### Conclusions.

For the first time, SCC*mec*-like elements have been identified in *Enterococcus*. The novel element was named enterococcus cassette chromosome (ECC) and shared characteristics like the insertion site downstream of *rlmH*, *att* site sequences, and variable gene content with SCC*mec*. We also show mobilization with the help of a conjugative *rep*_pLG1_ megaplasmid.

Cassette chromosome elements had previously been found only in the *Staphylococcus* genus. The existence of a similar element in *Enterococcus* suggests that cassette chromosome elements are more abundant than previously thought. Several resistance genes (toward methicillin, kanamycin, tobramycin, bleomycin, penicillins, heavy metals, tetracycline, macrolide, lincosamide, and streptogramin) have been found in SCC*mec* (44, 45), but only one ECC harbored a tetracycline resistance gene. Introduction of other clinically important resistance genes in the ECC element such as *mecA* in SCC*mec* may result in more spread and stability of this type of element due to antimicrobial selection.

The ECC gene content variability parallel results of Farrugia et al. (46) who found a family of GIs in *Proteobacteria* which were characterized by site-specific insertion in tRNA-dihydrouridine synthase A (*dusA*) by *dusA*-associated integrases (DAIs). The only universal features of these GIs were presence of DAIs and a consensus insertion sequence within the *dusA* gene, while the accessory genes within the GI varied extensively.

On the basis of the genetic contents in the studied ECC elements, we propose that they act as vehicles for exchange of genes in E. faecium. In SCC*mec* typing systems, accessory genes are located in the originally termed “junkyard” or “joining” “regions” (47). Little is known about the accessory genes in SCC*mec* and what effect they confer to their hosts in various environments. Accessory genes in general often seem to encode functions associated with peripheral functions thought to aid survival of bacterial populations in changing environments (48, 49).

Several others have indicated that genomic islands perform an adaptive evolutionary role for their hosts (50–52). Introduction into new ecological niches may be aided by gene acquisition and loss within these genomic sites. Understanding the underlying dynamics of such events is crucial to understand the evolution of their respective hosts, as well as the stability and dissemination of the individual GI itself.

The enterococci have already been shown to contain a vast array of mobile genetic elements. Here we add another layer of complexity to the E. faecium pan-genome through the discovery of an element with a variable gene content. Future endeavors connecting the genes of the mobilome by how they travel between MGEs such as ECC could shine light on the genetic connectivity of a highly recombinogenic species such as E. faecium.

## MATERIALS AND METHODS

### Bacterial strains, plasmids, and growth conditions.

The bacterial strains and plasmids used in this study are listed in Table 1. Escherichia coli strains were grown in Luria-Bertani broth or agar and E. faecium in brain heart infusion (BHI) broth or agar at 37°C unless specified otherwise.

The German clinical E. faecium ST17 UW1551 (53) was first partially plasmid cured by growth in novobiocin at 45°C overnight. After curing, the strain designated UWΔp showed a different plasmid profile (results not shown) visualized by gel electrophoresis of plasmid DNA isolated by alkaline lysis (54) and had lost resistance to vancomycin, gentamicin, and tetracycline. A chloramphenicol resistance-encoding gene (*cat*) was then inserted by a double crossover into an ORF encoding a hypothetical protein immediately downstream of *ccrAB_Ent_* using pTEX5501ts (55), resulting in strain UWECC::*cat*. The *rep*_pLG1_ helper plasmid originating from the clinical E. faecium isolate K60-19 was first mated into isolate BM4105-RF (56) and from there into UWECC::*cat*.

### Introduction of chloramphenicol resistance gene downstream of *ccrAB_Ent_*.

The gene replacement protocol described by Nallapareddy et al. (55) with minor modifications was used to insert a chloramphenicol acetyltransferase (*cat*) gene into the open reading frame (ORF1) downstream of *ccrAB_Ent_*. In brief, an 822-bp-long upstream region of ORF1 designated ORF1UpDel was amplified from genomic DNA from strain UWΔp by using the ORF1UpDel primers with restriction sites NheI and HindIII, respectively (Table S3). The PCR product was digested with NheI and HindIII and ligated to similarly digested pTEX5500ts, resulting in pORF1a. Subsequently, an 842-bp-long downstream region of ORF1 designated ORF1DnDel, was amplified using primers ORF1DnDel including restriction sites for PstI and PvuI, respectively. This PCR product was digested with PstI and PvuI and ligated to similarly digested pORF1a, resulting in pORF1b, which is pTEX5500ts with cloned ORF1 fragments flanking the *cat* gene. pORF1a and pORF1b were transferred into E. coli TOP10 cells (Invitrogen) for propagation and plasmid purification. pORF1b was introduced into strain UWΔp by electroporation to generate an insertion of *cat* into ORF1. Correct insertion of *cat* in ORF1 was checked by PCRs using primers for amplification of single and double crossovers (Table S3), by SmaI PFGE, Southern hybridization with *ccrB_Ent_* and Cm (*cat*) probes using protocols described by Sivertsen et al. (57), and DNA sequencing.

10.1128/mSphere.00402-18.3 TABLE S3 Primers used in this study. Download Table S3, PDF file, 0.05 MB. Copyright © 2018 Sivertsen et al. 2018 Sivertsen et al. This content is distributed under the terms of the Creative Commons Attribution 4.0 International license.

Genomic DNA from E. faecium was purified using the Qiagen genomic DNA kit (Qiagen). PCRs were performed with a Gene Amp PCR system 9700 thermal cycler (Applied Biosystems) using *Pfu* turbo polymerase (Promega). PCR products were purified using EZNA Cycle pure kit (Omega Bio-Tek Inc.). Plasmid DNA from E. coli was purified using the EZNA plasmid minikit I (Omega Bio-Tek Inc.) or Qiagen plasmid maxikit (Qiagen). Constructs were transformed into E. faecium by electroporation using a Gene Pulser II (Bio-Rad) by the method of Nallapareddy et al. (55).

### Filter mating and verification of transconjugants.

The filter mating protocol from Sivertsen et al. (57) was used with the following antibiotics and concentrations: chloramphenicol (Chl), 30 mg/liter; gentamicin (Gen), 300 mg/liter; rifampin (Rif), 20 mg/liter; fusidic acid (Fus), 10 mg/liter. For schematic presentation of experiments and which elements were transferred, see Fig. S1 in the supplemental material. All experiments were done using BHI agar. The presence of *ccrAB_Ent_* in strains was determined by primers FB and RB from Bjørkeng et al. detecting *ccrB* (21). The presence of the *rep*_pLG1_ plasmid was determined by primers *aac*(*6’*)*Ie-aph*(*2”*)*Ia* F and R detecting the HLGR determinant (54). PCRs specific to strains UWECC::*cat* and BM4105-RF were designed by identifying genes unique to each genome through Roary (58) comparisons. Primers are listed in Table S3. Transconjugants were further verified and characterized by the use of SmaI and S1 nuclease PFGEs, Southern hybridizations with Dig-labeled *ccrB_Ent_* and *aac*(*6’*)*Ie-aph*(*2”*)*Ia* probes.

### Genome sequencing.

Experimental isolates were cultured on blood agar overnight and a single colony was transferred to BHI broth and grown overnight. Genomic DNA (gDNA) was extracted using the Promega Wizard genomic DNA purification kit with the addition of 30 U mutanolysin in the lysis step. gDNA was sent to the Norwegian Sequencing Centre (NSC) (University of Oslo) where the 20-kb library preparation protocol and 6-kb cutoff BluePippin (Sage Sciences) size selection were done and sequenced with the Pacific Biosciences RSII sequencer using P6-C4 chemistry, 360-min movie time, and one SMRT cell per sample. Illumina sequencing was performed at the Genomics Support Center Tromsø, with Nextera 500 Illumina technology. For Oxford Nanopore sequencing, gDNA was purified using the Qiagen Genomic-tip 100/G kit (Qiagen) following the manufacturer’s protocol for Gram-positive bacterial samples, with 50 U mutanolysin added to the lysis mixture. The library was prepared using the rapid barcoding kit (SQK-RBK001) and sequenced on an R9.4 flow cell (FLO-MIN106), both supplied by Oxford Nanopore Technologies.

### Bioinformatic analyses.

Reads from Pac-Bio sequencing were assembled and polished at NSC using the HGAP v3 (Pacific Biosciences, SMRT Analysis Software v2.3.0) software (59). Unitigs were circularized by Minimus2 from the AMOS package (60), and *dnaA* (chromosome) or *repA* (plasmid) genes were set at the first nucleotide positions of unitigs using the circlator software (61), as well as closed with PCRs (data not shown). BM4105-RF Illumina and Nanopore data were combined in Unicycler v0.4.4 (62) and polished with Pilon v1.22 (63) after initial nanopore base calling with albacore v2.1.7, standard trimming by porechop v0.2.3 (https://github.com/rrwick/Porechop), removal of reads of <2 kb, downsampling to 1 gb, and Illumina data adaptor trimming and quality trimming (Q > 28) with Trim Galore! (https://www.bioinformatics.babraham.ac.uk/projects/trim_galore/). Nanopore reads were then mapped to the circularized genome using minimap2 (64) and processed by samtools (65) to confirm uniform coverage.

All E. faecium and E. faecalis genome assemblies available as of June 2016 and other enterococcal genomes available as of May 2018 (*n* = 1,478) were downloaded from NCBI. Searches for *ccrAB_Ent_* (UniProt accession nos. Q3Y3B0 and Q3Y3B1) were done with BLASTn and BLASTp. Perfect and imperfect repeats were identified using the NUCmer (v3.1) software (66) with a window size of 20 nt. Searches for *att* sites in enterococcal sequences was done with BLASTn-short. Pairwise alignment figures were created with EasyFig v.2.2.2. All ECC elements and novel genome sequences were annotated using prokka v1.11 (67) and further manual curation with BLASTp results. Transfer of *ccrAB_Ent_*-containing elements and surrounding regions were manually inspected in Artemis and Artemis Comparison Tool (68). ProgressiveMauve (69) from Mauve v.2.3.1 with standard settings was used to find common local colinear blocks and produce an alignment figure of *ccrAB_Ent_* elements. Gene clustering of *ccrAB_Ent_* elements were done with Roary (58) using standard settings. Phylogeny of E. faecium was produced by constructing a whole-genome alignment using Parsnp v.2.1.8 (70). Consensus motifs for *att* sites were produced at the MEME webpage (71). Functional annotation of ECC genes was done using the eggNOG mapper (72) and eggNOG database (73). To find ECC genes in E. faecium genomes, a representative gene of each gene cluster as determined by Roary v.3.6.8 was included in a custom nucleotide BLAST database in ABRicate (https://github.com/tseemann/abricate). ECC genes were found in E. faecium genomes with inclusion criteria defined as >75% DNA identity to include functionally equivalent genes and >95% coverage to exclude smaller genes.
