# Supplementary figures and images for: The Enterococcus Cassette Chromosome, a Genomic Variation Enabler in Enterococci
Source: mSphere. 2018 Nov 7;3(6):e00402-18. doi: 10.1128/mSphere.00402-18 (PMC6222049; doi:10.1128/mSphere.00402-18)

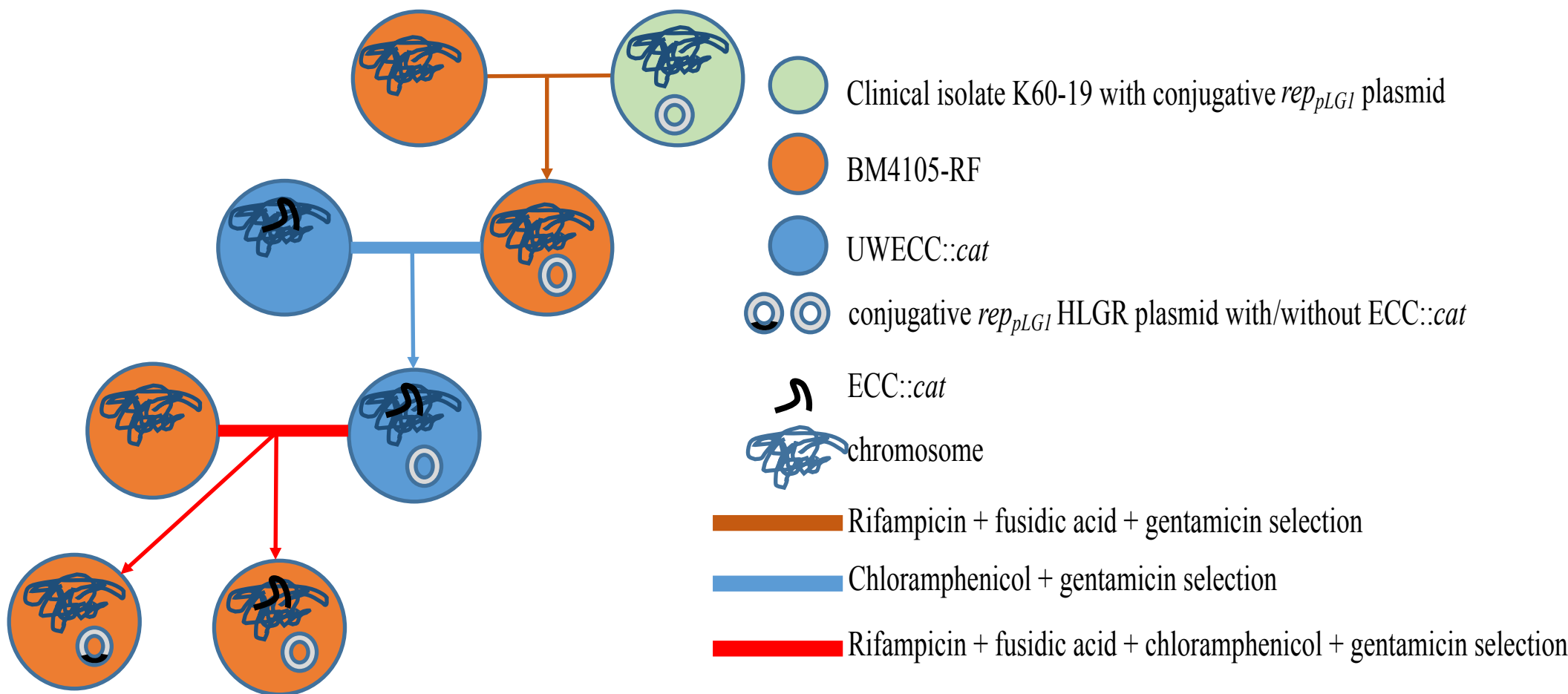

Supplement: FIG S1 [file sph006182686sf1.pdf]

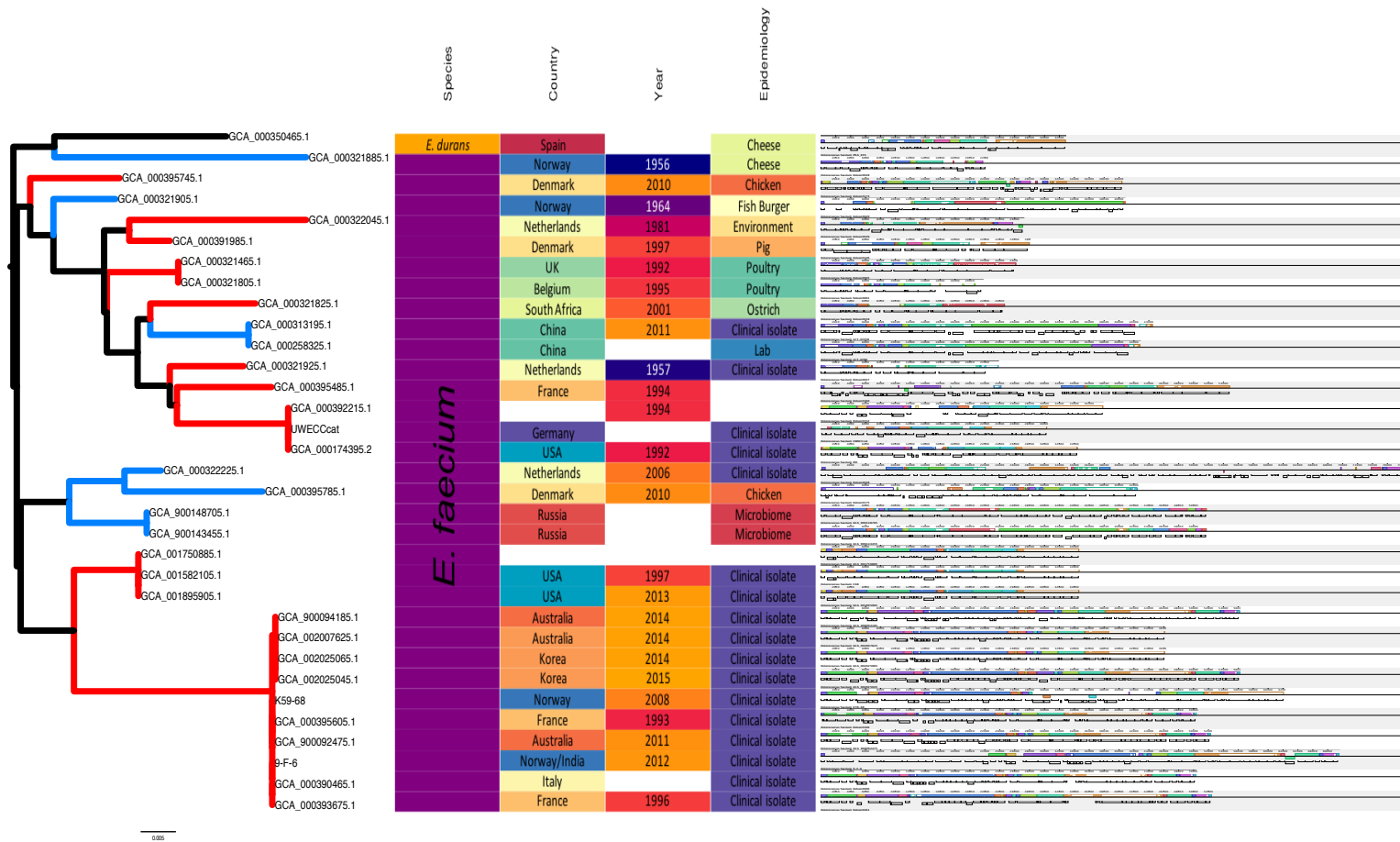

Supplement: FIG S2 [file sph006182686sf2.pdf]

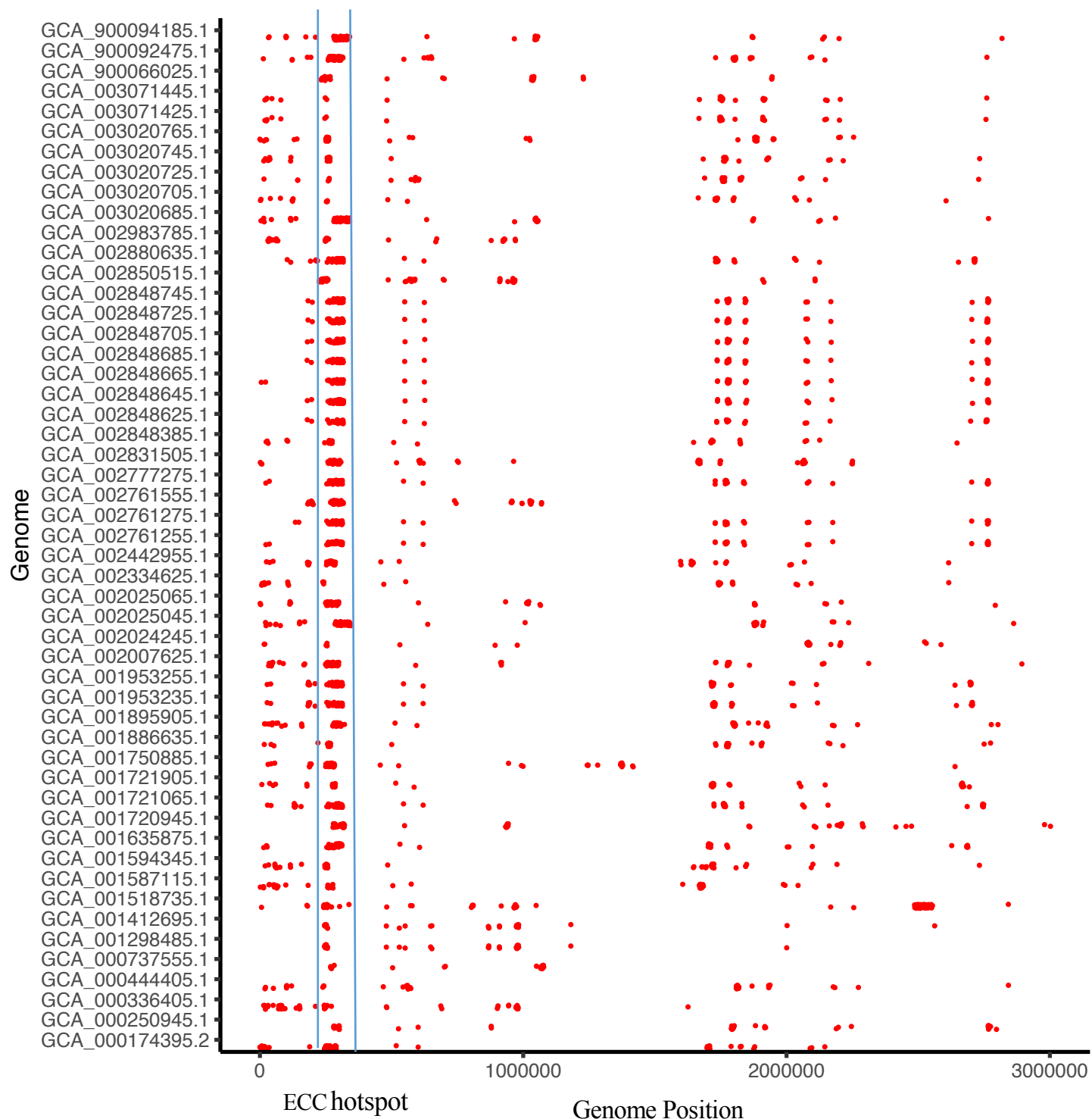

Supplement: FIG S3 [file sph006182686sf3.pdf]
