# Supplementary material for: The Enterococcus Cassette Chromosome, a Genomic Variation Enabler in Enterococci
Source: mSphere. 2018 Nov 7;3(6):e00402-18. doi: 10.1128/mSphere.00402-18 (PMC6222049; doi:10.1128/mSphere.00402-18)
Supplement: TABLE S3 [file sph006182686st3.pdf]

Primers for detection of the ECC and HLGR plasmid	Forward primer sequence 5'-3' *	Reverse primer sequence 5'-3'	Length in nucleotides	Reference
ccrB	ATTTGTCGCCGACCGATTAAG	ACGATACAAGGCTTTGATTGCT	690	21
aac(6')Ie-aph(2")Ia	CCAAGAGCAATAAGGGCATACC	ACCCTCAAAAACTGTTGTTGC	674	23
**Primers for detection of circular ECC**				
ECC*cat* circular	TCGAAGGGTTGGCATCTGGACCCGCT	TGCGGACCACGCGCCAAGAGAA	784	This study
DO circular	ACCCTGATGCTTTCACCAGA	CCTCCAGACTCCGAAGACAG	925	
K59-68 circular	ACCCTGATGCTTTCACCAGA	CCTCCAGACTCCGAAGACAG	924	
**Strain specific primers**				
menD (specific for UWECCcat)	TGGCGTTAAGGGTCCCGTGC	CCAACCCCGATTTGAGACTTTCC	1076	This study
cas9 (specific for BM4105-RF)	TTTTTGCTCGACTGAATGAA	CATAAACATTTTTCGCTTGG	618	This study
**Primers for cloning of ORF1 fragments into pTEW5500ts***				
ORF1UpDel (NheI/HindIII)	AATT<u>GCTAGC</u>GTGGCAGCGTTAAATGAAGAAATA	AATT<u>AAGCTT</u>AACCTGCAAAACAAAATAGACC	822	This study
ORF1DnDel (PstI/PvuI)	AATT<u>CTGCAG</u>CTAAGTCAGATAAAACGGAGAA	AATT<u>CGATCG</u>TGCTTGAACTTGACCAGAAT	842	This study
**Primers for checking single and double cross-overs**				
ORF1UpF and CmR	CGGGGTTGTTACGTTGTCCAG	AAAGCATTTTCAGGTATAGGTG	1611	This study, 53
CmF and ORF1DnR	GAATGACTTCAAAGAGTTTTATG	TTTATGTTCACTCTCCTTGTATGCT	1529	53, this study
ORF1UpF and ORF1DnR	CGGGGTTGTTACGTTGTCCAG	TTTATGTTCACTCTCCTTGTATGCT	3147	This study
5500-MCS-IF and 5500-MCS-IIR	GACAATACTGATAAGATAATATATC	GCCGATAACTAAACGAAATAAACG	Negative PCR if plasmid	53

* Resitriction sites are underlined
